# Porn or Partner Arousal? When It Comes to Romantic Relationships, Not All Sexual Arousal Is Equal: A Prospective Study

**DOI:** 10.1007/s10508-024-02985-4

**Published:** 2024-08-30

**Authors:** Nicholas J. Lawless, Gery C. Karantzas

**Affiliations:** School of Psychology, Deakin University, 221 Burwood Hwy, Burwood, Melbourne, VIC 3125 Australia

**Keywords:** Subjective sexual arousal, Pornography, Sexual satisfaction, Relationship quality, Relationship stability

## Abstract

Little is known about the role of subjective sexual arousal within romantic relationships. The current paper addresses this limitation using a prospective study design that investigates the direct associations between subjective sexual arousal induced by a romantic partner (partner arousal) and arousal induced by pornography (porn arousal) on changes in relational outcomes. A total of 309 participants who were in a current romantic relationship completed measures of partner and porn arousal as well as relational outcomes (i.e., sexual satisfaction and relationship quality and stability) at baseline (T1) and completed a second assessment of relational outcomes 2 months later (T2). Partner arousal was found to have no significant associations with changes in relational outcomes; however, porn arousal was associated with significant declines in sexual satisfaction and relationship quality and stability over a 2-month period. This paper is the first to examine how feeling sexually aroused by porn may impact relational outcomes for those in romantic relationships.

**Supplementary Information:**

The online version contains supplementary material available at 10.1007/s10508-024-02985-4.

## Introduction

Subjective sexual arousal (i.e., the cognitive-affective experience of being sexually excited; Basson, 2002; Janssen, 2011; Sierra et al., 2019) is thought to play an important role when it comes to sexual and relationship functioning and stability in romantic relationships (Lawless et al., 2022). Specifically, when a person feels sexually aroused by their partner (partner arousal), they are likely to experience not only physiological arousal but also increased motivation to engage in, and experience, satisfying sexual activity (Both et al., 2004; Walton et al., 2016). Furthermore, partner arousal is likely to motivate a person to engage in relationship maintenance behaviors which bolster the likelihood of future sexual activity, and these behaviors are also likely to enhance relationship quality such as relationship commitment, emotional intimacy, and feelings of love and trust between romantic partners (Birnbaum & Finkel, 2015; Gillath et al., 2016). Therefore, feeling sexually aroused by a romantic partner is likely to predict positive changes to the sexual and relational well-being and stability of people’s romantic relationships.

However, a romantic partner is not the only stimulus that may induce feelings of sexual arousal. For instance, using pornography (porn) is known to trigger feelings of sexual arousal (porn arousal; Emmers-Sommer, 2018; Grubbs et al., 2019). Porn use is common in romantic relationships, with research finding that approximately 70–80 percent of men and 35–60 percent of women in romantic relationships use porn every year (Willoughby & Leonhardt, 2020; Willoughby et al., 2016). The high frequency of porn use in couples raises important questions as it relates to subjective sexual arousal and relationship functioning. First, to what extent is porn arousal associated with increases or decreases in sexual and relationship well-being over time? Second, how do the effects of porn arousal compare to the effects of partner arousal? In the current paper, we answer these questions using a prospective study design.

### The Sexual Behavioral System

The functioning of the sexual behavioral system centers around a primary goal. This primary goal (at least in heterosexual individuals) is successful conception through the enactment of sexual intercourse (Birnbaum, 2014; Butler, 2019). However, the system also entails a series of subgoals that are geared toward experiencing pleasurable and satisfying sex as well as engaging in relationship maintenance behaviors that strengthen the stability and quality of people’s romantic relationships (Gillath et al., 2016). Therefore, as subjective sexual arousal is a critical input into the activation of the sexual behavioral system (Janssen, 2011; Janssen et al., 2000), the nature of the sexual stimulus that induces this arousal is likely to have implications for how sexually satisfying people experience their romantic relationships, and the relationship quality and stability of their relationships over time.

### Sexual and Relational Outcomes of Partner Arousal

Given the goal-directed functioning of the sexual behavioral system, partner arousal should guide the behavior of individuals in ways that yield positive sexual and relationship outcomes over time. Specifically, partner arousal should enhance sexual motivation, physiological sexual arousal (e.g., penile erection in men and vaginal tissue swelling/lubrication in women), and the frequency of sexual activity with a romantic partner (Both et al., 2004; Walton et al., 2016). As a result, partner arousal should be associated with increases in sexual satisfaction over time.

When it comes to relationship outcomes, partner arousal should demonstrate an increase in relational stability and the quality of one’s romantic relationship over time. Enhancing relational quality and stability by fostering aspects of relationships such as emotional intimacy, commitment, trust, and love is likely to increase opportunities for sexual activity, thus achieving the function of the sexual system (Gillath et al., 2016). Hence, partner arousal is likely to be associated with greater relationship quality and stability over time.

### Sexual and Relational Outcomes of Porn Arousal

The interplay between porn arousal, the functioning of the sexual behavioral system, and its implications for sexual and relational outcomes is likely to be more complicated than for partner arousal. One possibility is that porn arousal may be positively associated with sexual and relationship outcomes. This is because sexual behaviors are likely to be dictated (at least partially) by opportunities for sexual activity (Both et al., 2004). Within the context of romantic relationships, these sexual opportunities are most often with a romantic partner due to their close proximity and because of the existing sexual history between couple members. Therefore, when feeling sexually aroused by porn, the operation of the sexual behavioral system may be reoriented toward the romantic partner as a way to satisfy the individual’s sexual needs and desires (Kohut et al., 2017). Furthermore, porn arousal may promote relationship maintenance behaviors that increase relationship quality and stability over time, because the maintenance of a positive relationship atmosphere enhances the probability of sexual opportunities with one’s romantic partner in the future (Gillath et al., 2016).

In contrast, another perspective is that porn arousal may be negatively associated with sexual and relationship outcomes. Given that sexual arousal is considered to orient a person’s attention, motivation, and behavior toward the sexual target of the arousal (Janssen et al., 2000; Salonia et al., 2010), porn arousal may direct a person’s energy and effort toward porn use rather than toward sexual activity and relationship maintenance behaviors with one’s partner. Thus, porn arousal may attenuate sexual satisfaction and relationship quality and stability over time.

### Research into Subjective Sexual Arousal and Romantic Relationships

There exists a dearth of research investigating the role of subjective sexual arousal in romantic relationships, and only one study to date has examined the extent to which partner arousal and sexual arousal by a stimulus other than one’s partner were associated with sexual and relationship well-being. A dyadic study of heterosexual couples by Lawless et al. (2022) found that, for both men and women, partner arousal was associated with greater relationship quality and sexual satisfaction, whereas non-partner arousal was associated with poorer relationship quality and sexual satisfaction.

Although this study provides some important initial insights into the role of subjective sexual arousal in romantic relationships, the study was cross-sectional in nature and used brief assessments of sexual arousal and sexual satisfaction. More importantly, the study did not directly assess porn arousal. That is, the assessment of sexual arousal induced by stimuli other than one’s romantic partner was general in nature. The implication of this is that respondents may have answered questions about non-partner arousal by recalling stimuli other than porn, such as an attractive work colleague or acquaintance at a social function.

Therefore, when it comes to understanding the sexual and relational outcomes of porn use, it is necessary to draw on evidence beyond the study of subjective sexual arousal. Yet despite decades of research, the effect of porn use on sexual and relational outcomes remains controversial. There exists a large proportion of studies to suggest that porn use is negatively associated with relationship outcomes such as sexual and relationship satisfaction (e.g., Doran & Price, 2014; Maas et al., 2018; Morgan, 2011; Sun et al., 2016; Wright et al., 2018). To illustrate, a meta-analysis by Wright et al. (2017) that included 50 studies from 10 countries (comprised of more than 50,000 participants) found that porn use was associated with significantly lower sexual and relationship satisfaction for men. Nonetheless, there are studies that suggest that porn use has either no association or a positive association with sexual and relationship outcomes. For example, a large qualitative study found that many porn users believed that using porn had no negative consequences for their romantic relationship (Kohut et al., 2017). Kohut et al. also noted that participants reported positive impacts of porn use on their romantic relationship more frequently than negative effects, which, they argued, corresponded with the results of previous research (Grov et al., 2011).

Irrespective of these mixed findings, what is clear is that no study to date has investigated the subjective sexual arousal aspect of porn, and whether this plays a critical role in understanding the effects of porn use on sexual satisfaction and relationship quality. Furthermore, the majority of work investigating the effect of porn use on sexual and relational outcomes has been cross-sectional in nature. Though recent longitudinal studies have produced mixed findings (Vaillancourt-Morel et al., 2020, 2023), for the most part, the few longitudinal studies that have been conducted suggest that porn use may be predictive of negative downstream effects on relationship outcomes (see Grubbs & Kraus, 2021 for a review; also, Muusses et al., 2015). Nevertheless, the limited longitudinal research to date has ushered calls for future research to investigate the relational outcomes of porn use over time (Campbell & Kohut, 2017; Grubbs & Kraus, 2021). The state of the field is such that it is unclear as to the extent that porn use, and specifically its sexually arousing properties, is associated with changes in relational outcomes over time. To address these limitations, the current study employed a prospective study design to investigate the extent to which partner and porn arousal were associated with changes in sexual satisfaction, relationship quality, and relationship stability over a 2-month period.

### Hypotheses

Given existing theory and available evidence, three hypotheses were derived. The first hypothesis related to the associations between partner arousal (measured at baseline) and changes in sexual and relational outcomes over a 2-month period. Hypotheses two and three were competing hypotheses related to the associations between porn arousal (measured at baseline) and changes in sexual and relationship outcomes. We outline these hypotheses in turn. (1) Partner arousal will be associated with increases in relationship quality, relationship stability, and sexual satisfaction over time. (2a) Porn arousal will be associated with increases in relationship quality, relationship stability, and sexual satisfaction over time. (2b) Porn arousal will be associated with decreases in relationship quality, relationship stability, and sexual satisfaction over time.

## Method

### Participants

A general community sample made up of 309 individuals (130 males, 159 females, and 20 other) were recruited to participate in the study, ranging from 18 to 72 years of age (*M* = 31.49, SD = 10.27). Of those, 66% were heterosexual and 25% were bisexual, while 9% reported another sexual orientation (e.g., gay, lesbian, and pansexual). Participants had been in a relationship with their current partner for over 7 years on average (*M* = 7.04, SD = 7.50), and 91% reported being in a monogamous relationship while 9% were in a non-monogamous relationship. In terms of having children, 29% reported having at least one child, and 71% reported having no children. Participants’ status of their current relationship was reported as follows: 42% steady dating, 17% de facto (living together for 2 years or more, but not engaged or married), 6% engaged, and 34% married. Over half (59%) of the participants were living with their current partners, 28% did not live with their partner, while 13% reported being in a long-distance relationship. Participants were White/European (65%), Hispanic (16%), African (12%), and Asian (7%). Seventy-eight percent had completed further education post-high school, while 22% had completed high school. The majority of participants were employed (94%), and 6% were unemployed. Most of the sample (83%) reported using porn in the past month, while 17% had not. Finally, 91% experienced no physical difficulties when engaged in sexual activity. However, 9% said that they experienced pain or lack of physical response during sexual activity as a direct result of one or more medical conditions affecting their body.

### Measures and Procedure

Participants were recruited online via Reddit. To participate, people needed to be at least 18 years of age, have been in their current romantic relationship for at least 6 months, have previously had sex with their current partner, and be fluent in English. The study advertisement was posted on a wide range of Subreddits, including those related to pornography, relationships, sex, and psychology. The advertisement stated that the study was exploring the role of sexual arousal in romantic relationships, provided details regarding the inclusion criteria and what participation would entail, and informed prospective participants that upon completion of the study they would be entered into a draw to win one of three $200 gift cards. A link was also provided at the end of the advertisement, which took participants to the study’s Plain Language Statement (PLS). The PLS was a comprehensive document containing all the study details such as aims and procedures.

After viewing the PLS and providing their consent, participants completed a 5-item screening questionnaire to ensure they met the inclusion criteria for the study. Those that completed the screening but did not meet the study criteria were redirected to a web page that thanked them for their interest and outlined the reason they were unable to participate. People who were eligible to participate were automatically redirected to complete a 20-min online survey (timepoint 1). This survey assessed a range of relationship variables including sexual arousal, relationship quality, relationship stability, and sexual satisfaction. In addition, the survey asked about an array of demographic variables which could act as potential confounders, including age, relationship length, relationship status (e.g., steady dating, married, etc.), cohabitation (whether participants lived with their partner), monogamy (were participants in a monogamous relationship with their partner), parental status (did they have any children), number of children at home, whether they were in a long-distance relationship with their romantic partner, and sexual dysfunction (whether they experienced pain or lack of arousal during sex due to a diagnosed medical condition). Participants also provided an email address, and 2 months after completing the survey at timepoint 1, participants received an email with a link and an invitation to complete the online survey a second time (timepoint 2). This survey was almost identical to the first survey, minus the demographic questions, and with the addition of a 20-item social desirability scale (specifically the Bidimensional Impression Management Index developed by Blasberg et al., 2014).

#### Partner Arousal

Partner arousal was assessed using a 5-item measure that was adapted from the Affective Sexual Arousal Scale (Mosher et al., 1988) and the Partner-Specific Sexual Liking and Sexual Wanting Scale (Krishnamurti & Loewenstein, 2012). The first three items asked participants to rate to what extent the following statements applied to them over the past month: “When I thought about having sex with my partner, I felt turned on,” “I felt sexually excited by my partner,” and “When I looked at my partner, I felt sexually aroused.” These three items were rated from 1 (*not at all*) to 7 (*extremely*). The final two items then asked participants how often they have felt the following over the past month: “Sexually aroused by your partner when you *were not* having sex” and “Turned on by your partner *whilst* having sex.” These two items were rated on a scale ranging from 1 (*never*) to 7 (*more than once a day*). The five items were averaged to create an overall score of partner arousal with higher scores indicative of greater subjective sexual arousal induced by one’s romantic partner. The measure was found to have good internal consistency (Cronbach’s alpha = 0.90). Immediately prior to completing this measure, participants were presented the following definition: “For the purposes of this study, sex is defined as intimate physical contact with your partner involving more than kissing. This includes things such as caressing breasts/chest, genital contact, oral sex, and intercourse.”

#### Porn Arousal

Porn arousal was assessed with a 5-item scale that was designed to mirror the measure of partner arousal described above. The first three items asked participants to rate to what extent the following statements applied to them over the past month: “When I thought about using pornography, I felt turned on,” “I felt sexually excited by pornography,” and “When I used pornography, I felt sexually aroused.” These three items were rated from 1 (*not at all*) to 7 (*extremely*). The final two items asked participants how often they have felt the following over the past month: “Sexually aroused by pornography when you *were not* using it” and “Turned on by pornography *whilst* using it.” These two items were rated on a scale ranging from 1 (*never*) to 7 (*more than once a day*). The five items were averaged to create an overall score of porn arousal with higher scores indicative of greater subjective sexual arousal induced by porn. This measure was also found to have good internal consistency (Cronbach’s alpha = 0.91). Immediately prior to completing this measure, participants were presented the following definition: “USING PORNOGRAPHY means to intentionally look at, watch, read or listen to sexually arousing material [pictures, videos, films, written text or audio] which depicts nudity and/or explicit sexual behavior” (Lawless et al., 2023).

#### Relationship Quality

Relationship quality was measured using the 6-item short form of the Perceived Relationship Quality Components questionnaire (Fletcher et al., 2000). This scale consists of the following items: “How satisfied are you with your relationship?” “How committed are you to your relationship?” “How intimate is your relationship?” “How much do you trust your partner?” “How passionate is your relationship?” and “How much do you love your partner?” All items were rated on a 7-point scale ranging from 1 (*not at all*) to 7 (*extremely*). Relationship quality was computed by averaging all the items to create a global score, with higher scores indicative of greater relationship quality (Cronbach’s alpha = 0.87).

#### Relationship Stability

Relationship stability was assessed using the 5-item dissolution consideration subscale from the Romantic Relationship Commitment and Leave Behavior Scale (VanderDrift et al., 2009). Participants rated the extent to which they had experienced the following statements over the past month: “I have been thinking about ending our relationship,” “More and more it comes to mind that I should break up with my partner,” “I find myself wishing that my partner and I weren’t romantically involved,” “I have been close to telling my partner that I want to end our romantic relationship,” and “I have told people other than my partner that I might end my romantic relationship.” The scale ranged from 1 (*do not agree at all*) to 9 (*agree completely*). All items were reverse scored before averaging to create a global score, such that higher scores were indicative of greater relationship stability (Cronbach’s alpha = 0.93).

#### Sexual Satisfaction

Sexual satisfaction was measured using the 6-item sexual satisfaction scale taken from the Quality of Sex Inventory (Shaw & Rogge, 2016). Participants were instructed to think about the past month and rate items relating to their sex life using a scale ranging from 0 (*not at all true*) to 5 (*completely true*). Items were as follows: “My sex life is fulfilling,” “I am happy with my sex life with my partner,” “My partner really pleases me sexually,” “I am satisfied with our sexual relationship,” “I am happy with the quality of sexual activity in our relationship,” and “Sexual activity with my partner is fantastic.” Sexual satisfaction was derived by averaging all the items to create a global score, with higher scores indicative of greater sexual satisfaction (Cronbach’s alpha = 0.96).

### Statistical Analysis

Of the 309 participants who entered the study, 249 completed assessments at both time points resulting in a participant attrition rate of 20.5%. Given this, we used full information maximum likelihood as our approach for handling missing data. For the primary analysis, a path analytic model was tested using AMOS 28, and the model was estimated using maximum likelihood estimation. As part of this model, all three outcomes—changes in sexual satisfaction, relationship quality, and relationship stability—were regressed onto both partner arousal and porn arousal. We also controlled for frequency of porn use in our analyses. The correlations between the three outcome variables were also freely estimated. Residual change scores were derived as an index of change from T1 to T2 for the three outcome variables. The residual change scores were derived by regressing the T2 score onto the T1 score for each outcome, respectively.^1^ Thus, the derivation of these residualized autoregressive estimates inherently control for the effects of T1. Therefore, the variability that is unaccounted for by the prior time point remains, and it is this variability that is considered to reflect change (Castro-Schilo & Grimm, 2018).^2^ Specifically, positive scores reflect increases in outcome variables over time while negative scores reflect decreases in outcome variables over time.

Model fit was evaluated using Hu and Bentler’s (1999) guidelines such that a Comparative Fit Index (CFI) and Tucker–Lewis Index (TLI) ≥ 0.95, Root-Mean-Square Error of Approximation (RMSEA) ≤ 0.05, and Standardized Root Mean Residual (SRMR) ≤ 0.06 are indicative of a very good fitting model. A priori power to detect a small effect size (*β* = 0.01) within the path model was estimated according to Satorra and Saris (1985). Detecting a small effect size for a near saturated model (df = 1) with a power of 0.80 required a sample size of 80. Thus, our study sample well exceeded the minimum sample size required to test our model.

Prior to testing the model outlined, preliminary analyses were conducted to determine the effects of 10 potential confounds: age, relationship length, relationship status, cohabitation, monogamy, parental status, number of children at home, long-distance relationship, sexual dysfunction, and social desirability (specifically impression management). The only confounding variable that was found to have any association with change in outcomes over the 2-month period was participants who reported being in a monogamous (versus non-monogamous) relationship. This confound was positively associated with change in relationship quality only, and the inclusion of this confound did not alter the pattern of associations between partner and porn arousal with changes in sexual and relationship outcomes. Given this, no confound variables were included in the primary analysis.

## Results

Descriptive statistics and correlations between the study variables as assessed at timepoints 1 and 2 are presented in Table 1. On average, participants reported moderate to high partner arousal and moderate porn arousal at T1 (baseline). Furthermore, participants reported high relationship quality and stability, and moderate to high sexual satisfaction across T1 and T2. Partner arousal and porn arousal were not significantly correlated. Partner arousal was found to be positively and significantly correlated with sexual satisfaction, relationship quality, and relationship stability at T1 and T2, whereas porn arousal demonstrated significant negative associations with sexual satisfaction and relationship stability at T2.^3^ All outcome variables (sexual satisfaction, relationship quality, and relationship stability) were moderately to highly correlated with one another at T1 and at T2. In addition, each outcome variable demonstrated moderate to high stability from T1 to T2.

**Table 1:** Zero-order correlations and descriptive statistics

	1	2	3	4	5	6	7	8	9	
1. T1 Partner arousal	–									
2. T1 Porn arousal	.07	–								
3. T1 Relationship quality	.64**	− .06	–							
4. T1 Relationship stability	.34**	− .03	.61**	–						
5. T1 Sexual satisfaction	.66**	− .08	.73**	.38**	–					
6. T2 Relationship quality	.59**	− .10	.81**	.53**	.61**	–				
7. T2 Relationship stability	.30**	− .15*	.56**	.66**	.36**	.74**	–			
8. T2 Sexual satisfaction	.58**	− .18**	.72**	.45**	.79**	.80**	.57**	–		
9. T1 Frequency of porn use	− .02	.74**	− .12**	− .08*	− .19**	− .16*	− .11	− .16**	–	
*M*	4.93	3.88	6.00	7.98	3.55	5.93	7.89	3.50	3.62	
SD	1.48	1.54	1.09	1.87	1.48	1.23	1.98	1.52	1.75	
Scale range	1–7	1–7	1–7	1–9	0–5	1–7	1–9	0–5	1–7	

*p < .05 and **p < .01

**Fig. 1:**
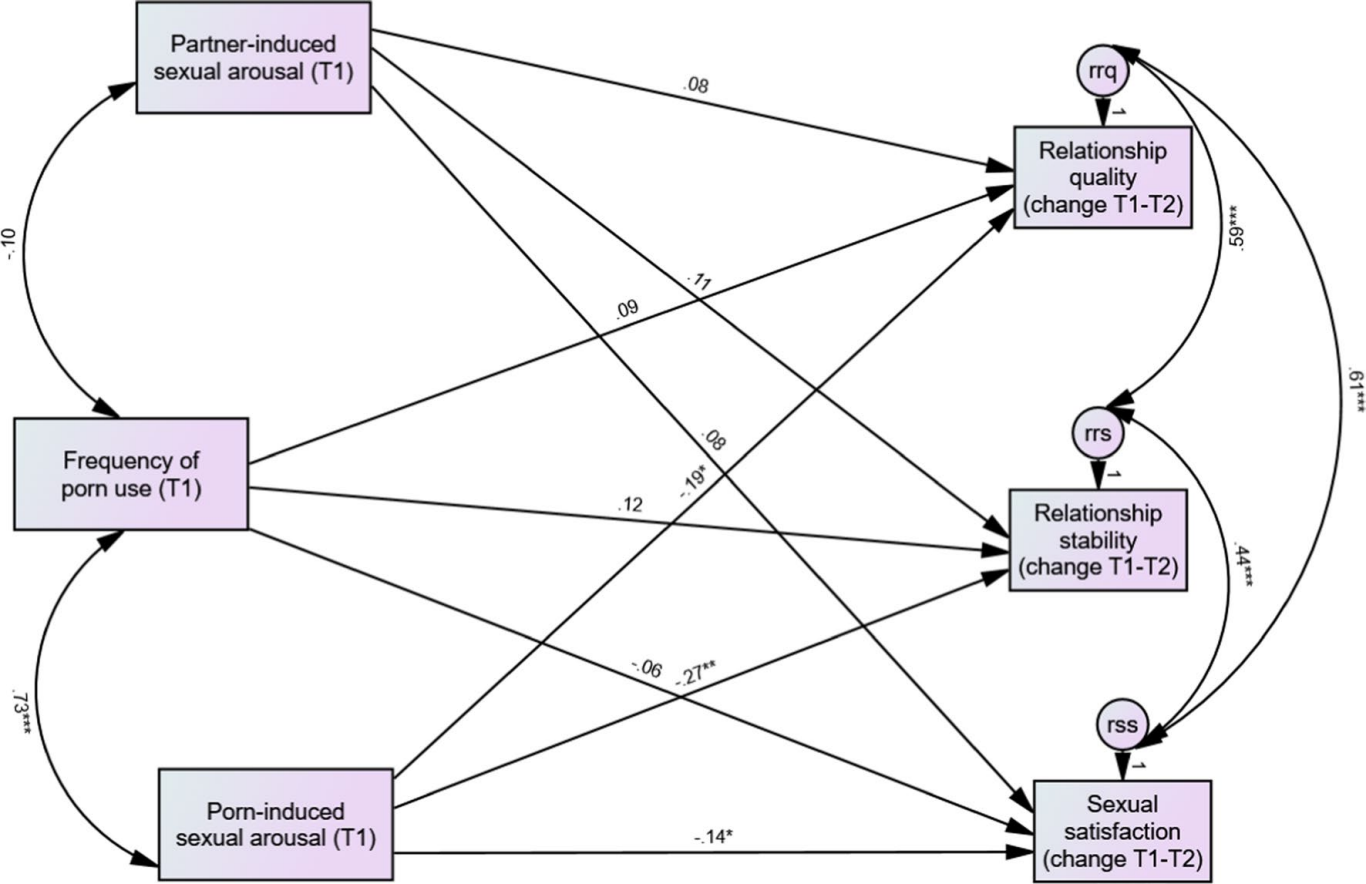
Path analysis of subjective sexual arousal and changes in relationship outcomes. *p < .05 and **p < .01. Note: rrq = error term relationship quality; rrs = error term relationship stability, and rss = error term sexual satisfaction. Path coefficients are standardized regression estimates (the unstandardized estimates for the path coefficients are reported in Supplement 1)

The primary analysis revealed that our path model demonstrated excellent fit to the data *χ*^2^(1) = 2.01, *p* = 0.16, CFI = 1.00, TLI = 0.95, RMSEA = 0.06, and SRMR = 0.04. As shown in Fig. 1, partner arousal was not significantly associated with change in relationship quality, relationship stability, or sexual satisfaction over the 2-month period. In contrast, porn arousal was significantly associated with reductions in all three outcomes over the 2-month period. Moderate associations were found between changes in outcomes variables.

## Discussion

This study used a prospective design to understand the role of subjective sexual arousal by one’s partner (partner arousal) and by porn use (porn arousal) on changes in sexual satisfaction and relationship quality and stability over time. In doing so, this study is the first to report on the relational changes associated with the sexually arousing properties of two contrasting sexual stimuli—a romantic partner and pornography, thereby extending on the previous research that focuses on frequency of porn use but has neglected to explore the sexually arousing properties of porn use. We derived three hypotheses based on theory regarding the functioning of the sexual behavioral system (Gillath et al., 2016) and through the examination of the limited research on sexual arousal and relational outcomes in romantic relationships (Lawless et al., 2022).

In contrast with our first hypothesis, partner arousal (as measured at T1) was not significantly associated with changes in sexual satisfaction and relationship quality and stability over a 2-month period. What do we make of this finding given that the very limited dyadic research to date as well as theory into the functioning of the sexual behavioral system would suggest that being sexually aroused by one’s partner should be positively associated with sexual satisfaction, positive evaluations of the quality of one’s romantic relationship, and relationship stability (Birnbaum & Finkel, 2015; Both et al., 2004; Lawless et al., 2022; Walton et al., 2016)?

Zero-order correlations demonstrated that partner arousal was positively associated with all three relational outcomes at T1 and T2, and the magnitude of these associations was consistent across the two time points. These zero-order associations support past cross-sectional research as to the contemporaneous relationship between partner arousal and relationship outcomes (Lawless et al., 2022). Our supplementary analyses modeling the associations between model variables at T1 (see footnote 3 and supplement 1) further confirm the associations between partner arousal and relational outcomes. However, it is when controlling for prior levels of sexual satisfaction and relationship quality and stability, that partner arousal does not appear to contribute to any more change in relational outcomes. One reason as to why partner arousal may not contribute to changes in relational outcomes is because of the high mean levels of these outcomes reported by the sample at T1 and T2 as well as the high stability of measures across the two time points. That is, the non-significant associations between partner arousal and relational outcomes may reflect ceiling effects. Indeed, if individuals already evaluated their romantic relationships as very high on sexual satisfaction and relationship quality and stability, there is little room for increases in outcomes.

In contrast, our findings for porn arousal suggest that despite people holding consistently high levels of sexual satisfaction, relationship quality, and relationship stability, porn arousal is indeed associated with declines across all outcomes. Of the two alternate hypotheses that we proposed with regard to the associations between porn arousal and changes in relational outcomes, our findings provide support for the prediction that porn arousal is associated with reductions (not increases) in sexual satisfaction and relationship quality and stability across a 2-month period.

Therefore, despite some evidence in support of the positive effects of porn on romantic relationships (e.g., Kohut et al., 2017), our findings suggest that this is not the case over time, even for those who report high relationship well-being. Rather, our findings are consistent with the majority of research that suggests that porn use is associated with negative relational outcomes (e.g., Doran & Price, 2014; Maas et al., 2018; Morgan, 2011; Sun et al., 2016; Wright et al., 2017, 2018). What is it about porn arousal though that compromises people’s relational outcomes?

Our findings suggest that sexual arousal by porn is not directed toward strengthening relational processes that can enhance the opportunities for satisfying sex and fostering relational behaviors that strengthen relationship quality and stability (Gillath et al., 2016). Although our findings cannot directly speak to whether porn arousal directs the functioning of the sexual system away from romantic partners toward increases in porn use, theory into the operation of the sexual system suggests that when sexually aroused, a person’s attention, motivation, and behavior are directed toward the stimulus target (Janssen et al., 2000; Salonia et al., 2010). Therefore, it may well be that being sexually aroused by porn increases people’s efforts toward porn use at the expense of their romantic relationship. At the very least, our findings suggest that porn arousal attenuates relationship well-being over time.

### Research Implications

Our findings are novel and have important research implications. First, our study addresses a neglected aspect of sex research, and that is the role of subjective sexual arousal in romantic relationships. It has been noted that sexual arousal must be considered in the context of relationships (Dewitte, 2014), and our research is an important attempt at addressing this research gap. Second, our study is the first to consider the impact of porn-induced subjective sexual arousal on relational outcomes and contrasts this assessment with an assessment of partner-induced subjective sexual arousal. Therefore, our findings can shed important insights into how the arousing properties of different sexual stimuli have implications for relational outcomes. Finally, our study addresses the dearth of research investigating the effects of porn use over time. Our findings suggest that although porn arousal may not demonstrate much by way of contemporaneous associations with relational outcomes (i.e., correlations between porn arousal and relational outcomes at T1), it is important to consider how arousal to porn may forecast or have downstream changes in relational outcomes.

### Limitations and Future Directions

Although the current study addresses a number of important gaps in research into the study of subjective sexual arousal and porn use in romantic relationships, the study is not without limitations. First, our assessment of relational outcomes is limited to two time points spanning 2 months. Therefore, we are unable to model nonlinear trajectories of relational outcomes, and to do so over an extended time frame. Future research that addresses this limitation can inform the field as to whether the negative associations between porn arousal and changes in relationship outcomes found in the present study extend well into the future, or whether negative effects abate over time.

Second, the study sample included participants that generally reported high sexual satisfaction and relationship quality and stability. This may in part be due to the convenience sampling approach we undertook by way of recruitment through Reddit. However, we did recruit across a range of Subreddits with the aim of capturing individuals who may be experiencing relationship difficulties and problematic porn use. Nevertheless, it is unclear whether the associations found in our study would generalize to those experiencing relationship distress. Future research would do well to investigate partner and porn arousal in those individuals who experience sexual dissatisfaction, poor quality, or have concerns about the future viability of their relationship. It may be that negative associations between porn use and relationship outcomes are larger for those who are experiencing relationship distress. Thus, investigations involving distressed samples are likely to be a particularly critical future direction in the study of partner and porn arousal on relationship well-being.

Third, despite the strengths of a longitudinal design, the study is unable to make causal conclusions. The study is also not immune to the inherent limitations of self-report measures (such as underreporting on the phenomena of interest), although the preliminary analyses suggested that social desirability (in this case, specifically impression management) did not influence the current findings. In addition, the sample was made up of individuals in romantic relationships. Given findings suggesting the interdependent nature of relationships, and specifically the effects of pornography use on couples (e.g., Vaillancourt-Morel et al., 2023), future research could use dyadic data to extend on the current findings.

Fourth, despite controlling for a variety of socio-demographic confounders, we did not control for religiosity. Given that some past research has found religiosity to be a factor in determining the effects of porn use (for a review, see Grubbs & Kraus, 2021), it may be important for future research to control for religiosity when examining the associations between porn arousal and relational outcomes.

Finally, the current study investigated the direct associations between partner and porn-induced sexual arousal and relationship outcomes over time. However, theory into the functioning of the sexual system suggests that there are likely to be a series of relational processes that operate to achieve subgoals of the sexual system. That is, there are likely to be several mediating variables that can help to explain the associations between sexual arousal and relational outcomes. Although the findings of the present study help to establish direct associations between arousal (by distinct stimuli) and relational outcomes, we can only assume as to the relational processes invoked by different sexually arousing stimuli. Therefore, an important avenue for future research is the study of mediators that directly speak to the processes theorized to align with the subgoals of the sexual system.

### Conclusion

The current study addresses a significantly under-studied area of sex and relationship research. Our findings suggest that porn arousal is associated with reductions in an array of relational outcomes over time. These findings have important implications for future research in understanding how feeling sexually aroused by porn can have negative downstream effects on relationship well-being.

## Supplementary Information

Below is the link to the electronic supplementary material.

Supplementary file1 (DOCX 18 KB)

Supplementary file2 (DOCX 138 KB)

## Data Availability

Data can be made available by a direct request to the author.

## Footnotes

1 We also ran the model regressing T2 scores onto T1 scores for the outcome variables while including the T1 partner and porn arousal variables. That model evidenced the same results; porn arousal was significantly negatively associated with all three outcomes at T2.

2 The model was also run employing a latent difference score approach to determine whether the modeling of latent change would result in different model associations on the basis of measurement error controls. The latent difference score model revealed the same associations as the residual change score approach. Given the parsimony of the residual change score approach, we report on the path model utilizing the residual change approach.

3 We also conducted a path analysis examining the association between all variables at T1 that were included in model tested in our primary analyses. This model was conducted to determine the extent to which partner arousal and porn arousal were contemporaneously associated with relationship quality, relationship stability, and sexual satisfaction. This model is presented in Supplement 1 and demonstrates that partner arousal was associated with all outcomes at T1, while porn arousal was not significantly associated with T1 outcomes.
