# Supplementary material for: Porn or Partner Arousal? When It Comes to Romantic Relationships, Not All Sexual Arousal Is Equal: A Prospective Study
Source: Arch Sex Behav. 2024 Aug 30;53(9):3451–60. doi: 10.1007/s10508-024-02985-4 (PMC11390763; doi:10.1007/s10508-024-02985-4)

**Supplement 1**

**95% Bias-corrected Confidence Intervals for Standardized Effects in Figure 1.**

**Lower-bound**

|  | **Porn arousal** | **Partner arousal** |
| --- | --- | --- |
| **Sexual satisfaction** | -.200 | -.030 |
| **Relationship stability** | -.320 | -.025 |
| **Relationship quality** | -.113 | -.020 |

**Upper-bound**

|  | **Porn arousal** | **Partner arousal** |
| --- | --- | --- |
| **Sexual satisfaction** | -.029 | .126 |
| **Relationship stability** | -.052 | .262 |
| **Relationship quality** | -.005 | .104 |

**Unstandardized estimates for path coefficients in Figure 1.**

|  |  |  | **Estimate** | **S.E.** | **C.R.** | **P** |  |
| --- | --- | --- | --- | --- | --- | --- | --- |
| Relationship quality | <--- | Partner arousal | .042 | .032 | 1.305 | .192 |  |
| Sexual satisfaction | <--- | Partner arousal | .045 | .041 | 1.101 | .271 |  |
| Sexual satisfaction | <--- | Porn arousal | -.113 | .038 | -2.939 | .003 |  |
| Relationship quality | <--- | Porn arousal | -.061 | .030 | -2.025 | .043 |  |
| Relationship stability | <--- | Partner arousal | .118 | .068 | 1.740 | .082 |  |
| Relationship stability | <--- | Porn arousal | -.182 | .063 | -2.888 | .004 |  |

**Variances for variables included in the path model in Figure 1.**

|  |  |  | **Estimate** | **S.E.** | **C.R.** | **P** | **Label** |
| --- | --- | --- | --- | --- | --- | --- | --- |
| **Partner arousal** |  |  | 1.980 | .178 | 11.136 | *** |  |
| **Porn arousal** |  |  | 2.282 | .205 | 11.136 | *** |  |
| **Relationship quality** |  |  | .506 | .045 | 11.136 | *** |  |
| **Relationship stability** |  |  | 2.257 | .203 | 11.136 | *** |  |
| **Sexual satisfaction** |  |  | .836 | .075 | 11.136 | *** |  |

**Cross-sectional path model for variables at T1 (Baseline)**


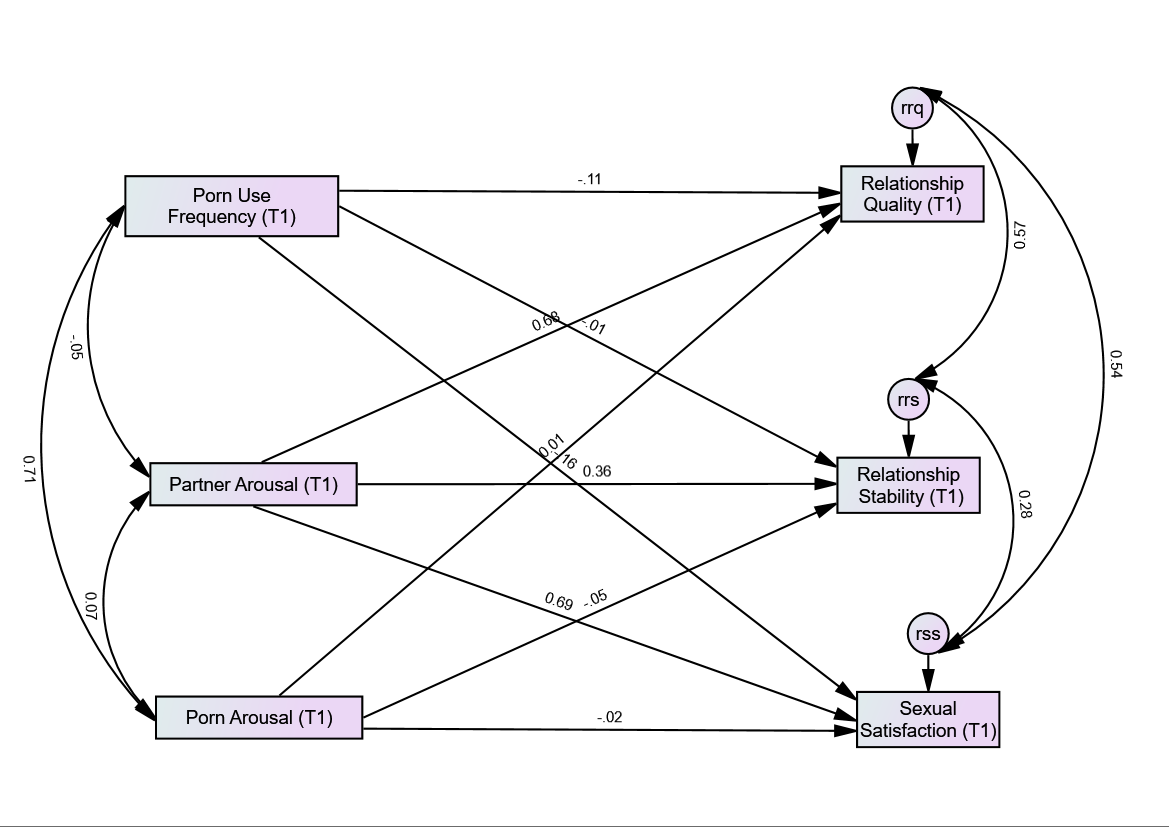

Supplement: Supplementary file 2 — Supplementary file2 (DOCX 138 KB) [file 10508_2024_2985_MOESM2_ESM.docx]
